# Sequential mediation analysis of physical activity, healthy diet, BMI, and academic burnout in the Pakistani educational landscape

**DOI:** 10.1038/s41598-024-58126-3

**Published:** 2024-04-02

**Authors:** Shazia Rehman, Abdullah Addas, Md Anisur Rahman, Muhammad Ali Shahiman, Zexuan Li

**Affiliations:** 1 Department of Psychiatry, National Clinical Research Center for Mental Disorders, National Center for Mental Disorders, The Second Xiangya Hospital of Central South University, Changsha, 410011 Hunan China; 2 Mental Health Institute of Central South University, China National Technology Institute on Mental Disorders, Hunan Technology Institute of Psychiatry, Hunan Key Laboratory of Psychiatry and Mental Health, Hunan Medical Center for Mental Health, Changsha, 410011 Hunan China; 3 Department of Civil Engineering, College of Engineering, Prince Sattam Bin Abdulaziz University, 11942 Al-Kharj, Saudi Arabia; 4 Landscape Architecture Department, Faculty of Architecture and Planning, King Abdulaziz University, P.O. Box 80210, 21589 Jeddah, Saudi Arabia; 5 Department of Accounting, Data Analytics, Economics and Finance, La Trobe University, Melbourne, Australia; 6 Department of Urology and Renal Transplantation, Benazir Bhutto Hospital, Rawalpindi Medical University, Rawalpindi, Pakistan

**Keywords:** Physical activity, Obesity, BMI, Healthy diet, Public health, Students, Academic burnout, Nutrition, Psychology, Health care

## Abstract

Research has indicated a negative impact of physical activity on academic burnout among students, however, there is a paucity of evidence about the underlying mechanism of this association in Pakistani students. The present research seeks to investigate the relationship between physical activity and academic burnout by investigating the potential mediating effects of adherence to the Mediterranean diet (MD) and body mass index (BMI). A sample of 596 students using a cross-sectional survey design was gathered from two public universities (Riphah International University and Mohammed Ali Jinnah University) in Rawalpindi, Pakistan from June to July 2022. The study participants were asked to fill out the Physical Activity Rating Scale-3, the Learning Burnout Scale, and the Test of Adherence to MD questionnaires. The study employed descriptive, bivariate, and path analysis through regression utilizing the SPSS software version 27. The findings demonstrated a negative correlation between academic burnout and BMI, physical activity, and adherence to the MD. The relationship between physical activity and academic burnout was mediated by BMI. Physical activity and academic burnout were inversely correlated, with adherence to the MD and BMI interacting as sequential mediators. The outcomes of this research have expanded our knowledge of the association between physical activity and academic burnout and have suggested crucial and appropriate strategies for addressing student academic burnout.

## Introduction

The term "academic burnout" describes a condition of extreme emotional, mental, and physical tiredness brought on by persistently high academic pressure. Feelings of extreme fatigue, cynicism or disengagement from scholastic pursuits, and a diminished sense of personal achievement are its defining characteristics^1^. Academic environments such as universities or schools, where people are under constant stress from performance expectations, academic demands, or an excessive workload, are the typical places where academic burnout happens. Individuals can exhibit avoidance behaviors, procrastination, or disengagement from academic responsibilities, such as skipping classes or showing disinterest in previously enjoyed academic activities and extracurriculars^2,3^. The phenomenon of academic burnout has been associated with adverse effects on physical and mental health, academic performance, and overall well-being^4,5^. Recognizing indicators of burnout and seeking appropriate assistance are imperative in effectively addressing this phenomenon. To effectively manage and prevent academic burnout, employing strategies such as prioritizing self-care, seeking academic support, establishing realistic goals, utilizing stress management techniques, and seeking professional assistance when necessary is essential.

The Coronavirus disease 2019 (COVID-19) pandemic has brought about significant changes to the global education sector, leading to an urgent transition to online learning and presenting unparalleled difficulties for students^6^. Research has indicated an increase in stress, anxiety, and burnout among students in the aftermath of COVID-19, underscoring the importance of investigating potential mitigating factors for these negative impacts^7,8^. This context establishes the foundation for examining the potential impact of physical activity on mitigating academic burnout among students in Pakistan. One of the notable issues pertains to the interruption of physical activities, which has garnered attention due to its potential influence on academic achievement^9^. The measures implemented in response to the COVID-19 pandemic, such as school closures, social distancing requirements, and lockdowns, have restricted students' opportunities to utilize sports facilities, participate in physical education classes, and engage in extracurricular activities^10^. The cumulative impact of these alterations leads to a decrease in students' physical activity levels, prompting concerns about potential consequences for their academic achievement.

There are lots of different theories that say physical activity is good for your academic performance. One of the most popular is called arousal and attention. This means that when you exercise, your body is more likely to be excited and you'll release more of the neurotransmitters that are involved in focusing and paying attention. This includes dopamine, which is linked to attention and focus^11,12^. Another theory says that exercise is good for your cognitive abilities because it helps you focus on both your cognitive and motor tasks at the same time. This could mean that exercise can help improve your executive function^13^. Physical activity is consistently linked with enhanced cognitive functioning, including attention, memory, and information processing. Research has shown that students who exercise regularly have better academic performance, including higher grades and standardized test scores^14,15^. These cognitive outcomes are particularly important in academic contexts, where sustained attention, efficient information processing, and memory recall are essential components of learning and academic success.

The exploration of the relationship between physical activity and academic performance holds significant implications for the improvement of educational practices. Incorporating physical activity into the school day, such as through physical education classes, active breaks, or extracurricular sports, has the potential to provide a comprehensive strategy for enhancing students' cognitive development and academic achievement. Policymakers and educators may wish to explore the integration of evidence-based physical activity programs as a means to enhance learning environments and foster the holistic well-being of students. In a nutshell, an abundance of theoretical frameworks and empirical data indicate a significant association between academic success and physical activity. Better academic results are a result of physical activity's neurobiological, psychological, and cognitive advantages. Understanding the complex interactions that exist between physical activity and cognitive function highlights the need to encourage students to lead active lifestyles to improve their physical health as well as their cognitive capacities and academic performance.

The Mediterranean diet (MD), widely acclaimed for its favorable impact on physical health, has recently attracted interest for its potential effects on mental well-being^16^. The MD is characterized by a high content of antioxidants, omega-3 fatty acids, and various nutrients that have been linked to enhanced cognitive function and psychological health^17^. Prior literature has examined the association between dietary intake and mental well-being, highlighting the significance of nutrition in alleviating manifestations of depression and anxiety^18,19^. The correlation between adherence to the MD, levels of physical activity, and academic burnout has received limited attention in current research^20–22^. Research has shown that maintaining the MD is associated with heightened energy levels and enhanced physical well-being. On the contrary, consistent physical activity may augment the beneficial impacts of the MD, establishing a synergistic interplay between dietary choices and exercise that supports holistic health^16,23^. The prospective influence of the MD on academic burnout represents a unique focus of investigation in this study. Academic burnout has been linked to a range of lifestyle-related factors^24^. Comprehending the relationship between adherence to the MD and its potential role as a mitigating factor against academic burnout is essential for the formulation and implementation of comprehensive interventions aimed at supporting students in their pursuit of academic achievement.

The existing literature highlights the individual relationships between physical activity, diet, BMI, and their impact on mental health and academic outcomes. However, there is a notable dearth of research that explores the interconnectedness of these factors. The current research aims to fill this lacuna by examining the sequential mediation of adherence to the MD and BMI in the association between physical activity and academic burnout. The objective of the present investigation is to explore the intricate mechanisms by which engaging in regular physical activity can potentially contribute to the improvement of students' mental health and academic achievement in the post-COVID-19 era. Subsequently, we posit the following hypotheses:

### Hypothesis 1

There exists a negative association between Physical activity and academic burnout.

### Hypothesis 2

Adherence to the MD plays a mediating role in explaining the relationship between physical activity and academic burnout.

### Hypothesis 3

BMI plays a mediating role in explaining the relationship between physical activity and academic burnout.

## Material and methods

### Participants and study design

A cross-sectional survey study was conducted employing a convenience sampling method, utilizing a single survey instrument within a single cohort. During June and July in the year 2022, a sample comprising 596 students from two public universities located in Rawalpindi, Pakistan, was collected for the investigation. The study participants' ages ranged from 18 to 26 years. The proportion of male students to female students was 49.23% and 507.7%, respectively.

### Eligibility criteria

Participants were eligible for the study if they were older than 18 and younger than 30 and had no diagnosed mental illness. Participants under the age of 18 were not allowed to participate in the study.

### Questionnaire

The following main scales were adopted to collect the information to run the analysis.

1. Participants' physical Activity based on intensity, duration, and frequency was evaluated through Physical Activity Rating Scale-3 revised by Liang^25^. The responses were recorded on a 5-point Likert scale, with the total score estimated by the product of physical intensity, duration, and frequency. The scale showed an internal consistency with an alpha coefficient of 0.87 for the current research.
2. The status of academic burnout was assessed through the Learning Burnout Scale for College Students^26^. This scale consisted of 20 questions in total that assess three distinct dimensions of academic burnout i.e., emotional exhaustion, and low personal achievement. The responses are recorded on a 5-point Likert-type scale, with higher scores indicating higher academic burnout. The scale showed an internal consistency with an alpha coefficient of 0.91 for the current research.
3. A scale consisting of sixteen dichotomous items i.e., Test of Adherence to Mediterranean Diet (KIDMED)^27^, was adopted to record the responses of participants which consisted of 16 dichotomous. The scale showed an internal consistency with an alpha coefficient of 0.86 for the current research.
4. BMI was assessed based on the self-administered response on body weight (kg) divided by squared height (m^2^)

### Ethical consent

The study was granted ethical approval from the Ethics Review Committee of Benazir Bhutto Hospital, Rawalpindi, Pakistan (March 2022/07613) and was conducted according to the guidelines and regulations of the Declaration of Helsinki and its later amendments. The participants provided their written informed consent to participate in this study.

### Data analysis

Descriptive and Pearson correlation analyses were utilized to estimate the associations among study scales. SPSS macro-PROCESS 4 with a 95% confidence interval and 5000 bootstrap iterations were performed to investigate the study hypotheses. A *p*-value of < 0.05 was considered to be significant statistically.

### Descriptive and pairwise correlation analysis

**Figure 1:**
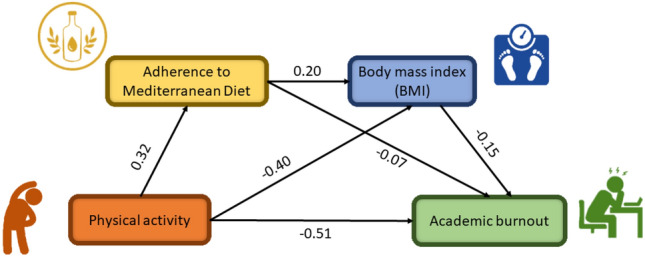
Bivariate correlation among study variables.

Table 1 presents the findings of descriptive and bivariate correlation analysis about the selected study variables. The findings of this study indicated a positive connection between physical activity and adherence to the MD, whereas, BMI and physical activity showed a significant negative association. Similarly, there was a positive correlation between adherence to the MD and BMI. Furthermore, it was found that engaging in regular physical activity and maintaining adherence to the MD was negatively associated with academic burnout. These organizations initially offered support for the proposed indirect effects. Figure 1 represents a graphical representation of bivariate correlations among study variables.

**Table 1:** Bivariate interconnections of the study variables.

	Mean ± SD	Academic burnout	Physical activity	BMI	Adherence to Mediterranean diet
Academic burnout	56.72 ± 9.68	1			
Physical activity	25.12 ± 11.08	**− **0.22**	1		
BMI	3.50 ± 1.26	**− **0.31***	-0.48***	1	
Adherence to Mediterranean diet	2.67 ± 0.98	**− **0.18**	0.35**	0.23**	1

***p < 0.001, **p < 0.01, *p < 0.05.

### Regression analysis

**Figure 2:**
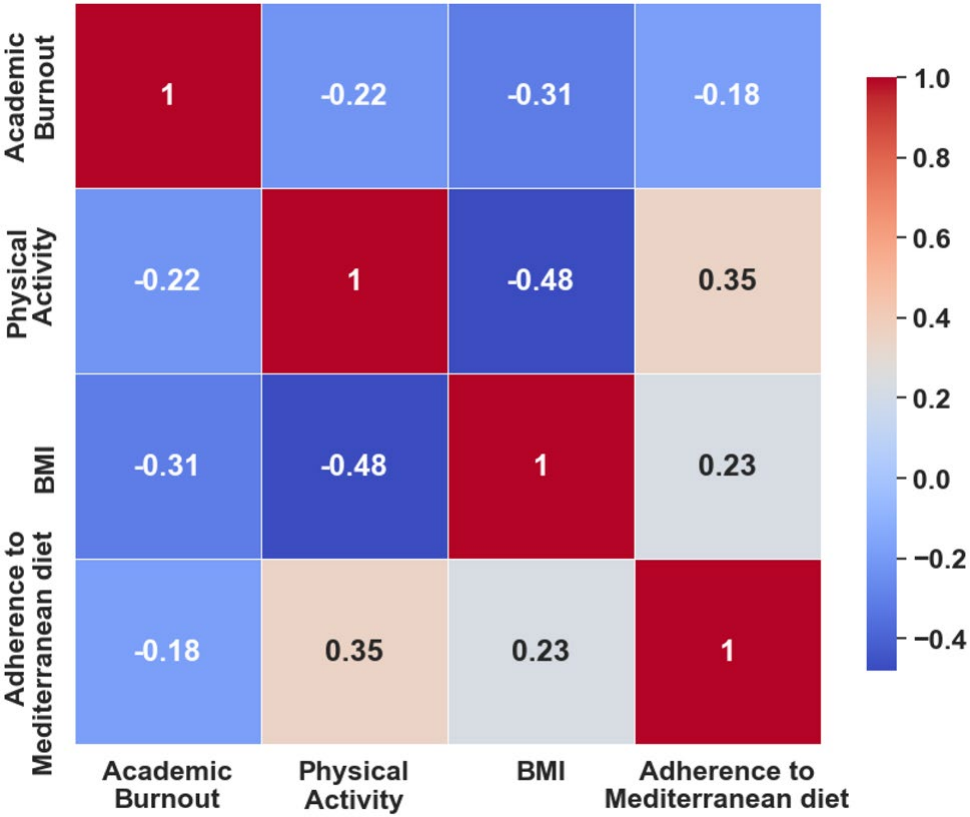
β-coefficient among study variables.

The findings of the regression analysis are presented in Table 2. The initial model revealed that there is a significant independent predictive relationship between physical activity and BMI (β = − 0.40, *p* < 0.001) and academic burnout. Both physical activity and BMI were found to be significant determinants of adherence to the MD, with statistical beta coefficients of 0.32 and 0.20, respectively. The study progressed to the third step where physical activity, BMI, and adherence to the MD were incorporated into the regression model. The findings indicated that physical activity, BMI, and adherence to the MD were significant predictors of academic burnout, with respective beta coefficients of − 0.51 (*p* < 0.001), − 0.15 (*p* < 0.001), and − 0.07 (*p* < 0.01) (Fig. 2). The aforementioned research findings indicate that physical activity exerts a partial mediating influence on academic burnout.

**Table 2:** Regression analysis results.

Regression equation		Fitting indices		Regression coefficient	
Response variable	Predictor variable	*R*^*2*^	*F*	$\beta$	*t*
BMI		0.14	62.17***		
	Physical activity			− 0.40	− 9.06***
$\mathrm{Adherence\,\,to}$ $\mathrm{Mediterranean\,\,diet}$		0.10	41.75***		
	Physical activity			0.32	3.95***
	BMI			0.20	14.35**
Academic burnout		0.37	83.06***		
	Physical activity			− 0.51	− 11.19***
	BMI			− 0.15	− 7.13***
	$\mathrm{adherence\,\,to\,\,Mediterranean\,\,diet}$			− 0.07	− 1.17**

**p < 0.01 and ***p < 0.001.

#### The serial mediation analysis

**Figure 3:**
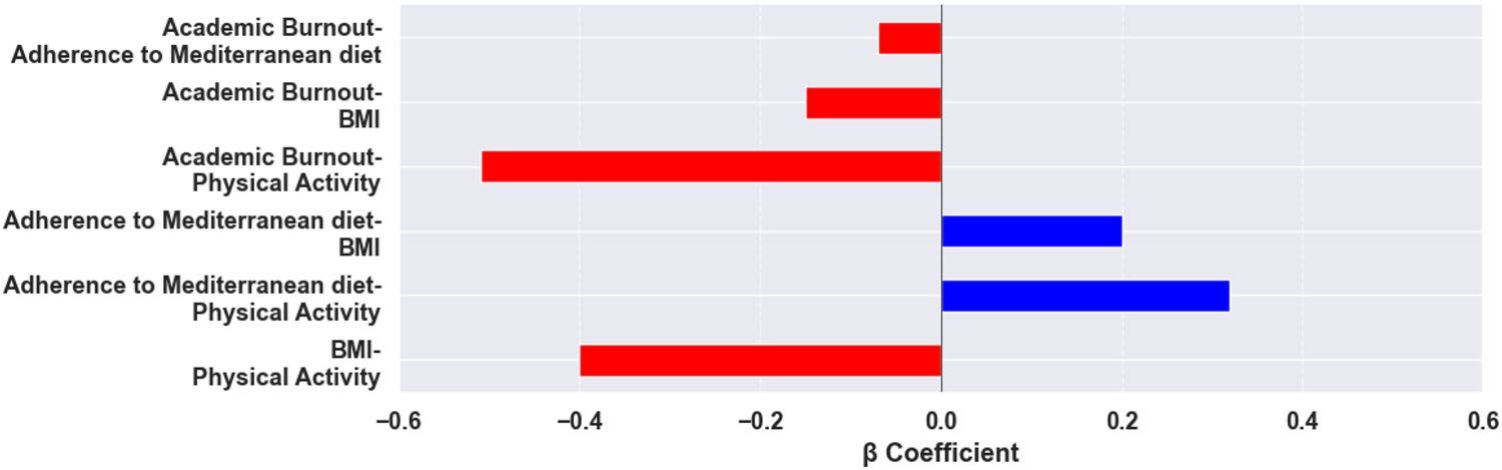
The path mediation analysis.

We estimated a sequential mediation model for the association between physical activity and academic burnout, adherence to the MD, and BMI using a bias-corrected nonparametric test with 95% confidence intervals (Table 3). The estimated direct effect of physical activity on academic burnout was observed to be 0.51, accounting for 85% of the overall effect. The study found that adherence to the MD, BMI, and engagement in physical activity were connected to the total indirect effect of 0.09 on academic burnout accounting for 15% of the overall effect. The mediating effect comprises two indirect pathways: one involving the relationship between physical activity and BMI, accounting for 10% of the total effect; and the other involving the indirect effects of physical activity on adherence to the MD, which in turn affects BMI and subsequently impacts academic burnout, representing 2% of the total effect. The schematic diagram for the mediation analysis of the study can be seen in Fig. 3.

**Table 3:** The results of the path analysis.

Path	Effect	SE	Boots 95% (lower–upper)	Outcome
Physical activity → ${\text{adherence\,\,to\,\,the\,\,Mediterranean\,\,diet}}$ → Academic burnout	− 0.02	0.02	− 0.03–0.04	Insignificant
${\text{Physical\,\,activity}}\, \to \,BMI\, \to \,$ Academic burnout	**− 0.06**	0.03	0.03–0.11	Significant
${\text{Physical\,\,activity}}\, \to \,{\text{adherence\,\,to\,\,Mediterranean\,\,diet}}\, \to \,BMI\, \to \,$ Academic burnout	**− 0.01**	0.02	0.01–0.09	Significant
Indirect effects	**− 0.09**	0.006	0.05–0.16	Significant
Direct effects (Physical activity → Academic burnout)	**− 0.51**	0.005	0.03–0.15	Significant
Total effect	**− 0.60**	0.03	0.58–0.66	Significant

The bold shows a significant effect based on Bootstrapped 95% confidence interval with 5000 iterations.

#### Explained variance and effect size

The assessment of the structural model was conducted with consideration of the explained variance and effect size criteria. The findings of this evaluation are demonstrated in Table 4. The aforementioned criteria were employed to evaluate the integrity and reliability of the structural model. The overarching model exhibited a moderate effect, as evidenced by an explained variance of 37%. We assessed the moderate effects of explicative variables in the associations between physical activity and adherence to the MD, physical activity, and BMI, and their relatively weak effect on academic burnout. The outcomes indicated that physical activity could potentially exert a significant influence on academic burnout, potentially mediated by adherence to the MD and BMI.

**Table 4:** The outcomes of explained variance and effect size.

Paths	Explained variance	Effect size
Physical activity $\to {\text{Adherence}}\;{\text{to}}\;{\text{Mediterranean}}\;{\text{diet}}$	0.14	0.22
Physical activity → BMI	0.10	0.29
${\text{Adherence}}\;{\text{to }}\;{\text{Mediterranean}}\;{\text{diet}}$ → BMI		0.10
Physical activity → Academic burnout	0.37	0.11
${\text{Adherence}}\;{\text{to }}\;{\text{the}}\;{\text{Mediterranean}}\;{\text{diet}}$ → Academic burnout		0.13
BMI → Academic burnout		0.09

### Ethics review statement

The study was granted ethical approval from the Ethics Review Committee of Benazir Bhutto Hospital, Rawalpindi, Pakistan (March 2022/07613) and was conducted according to the guidelines and regulations of the Declaration of Helsinki and its later amendments.

### Informed Consent Statement

Informed consent was obtained from all subjects involved in the study.

## Discussion

The present research empirically investigated the sequential mediating role of adherence to the MD and BMI on the potential correlations between physical activity and academic burnout. The regression analysis elucidated a sequential mediation model of the relationship between adherence to the MD and BMI. Our research demonstrated that physical activity was negatively associated with burnout among students. This implies that students who engage in consistent physical activity possess a lower risk of developing academic burnout. Moreover, these results align with earlier studies carried out within this area of research^28–30^. A plausible rationale could be that physical activity is linked to a considerable elevation in affirmative effect, resulting in enhanced productivity and less exhaustion during educational responsibilities^31^. Our hypotheses are supported by the considerable Pearson's pairwise correlation analysis that was found significant between BMI, academic burnout, physical activity, and adherence to the MD.

Our first hypothesis was supported by our findings, which showed that BMI served as a mediator in the association between physical activity and academic burnout. The beneficial effect of physical exercise on BMI was in line with other research suggesting that anxiety related to stressful events may change dietary behaviors, which in turn may alter a person's body composition^32–34^. In particular, anxiety will cause the parasympathetic nervous reflex to become active, which can speed up metabolism and stimulate appetite^35^. Furthermore, prolonged periods of stress have been associated with increased food intake, which may be explained by changes in the hypothalamus' ability to produce neurotransmitters that regulate one’s appetite^36^. Additionally, adherence to the MD and BMI were found to have a serial mediation impact in this research, thereby supporting our second hypothesis. Integrating therapeutic and preventative opinions it is crucial to emphasize two aspects regarding the significance of eating behaviors at this juncture. First and foremost, maintaining an adequate diet can help lower stress levels since it provides the human system with the necessary nutrients and prevents irregularities in neurotransmitter generation, which can exacerbate anxiety. Additionally, existing evidence indicates that the stressors themselves may contribute to maladaptive behaviors associated with the consumption of unhealthy foods^37^. Hence, there exists an enthusiasm for aligning actions with the requirements of the academic framework, aiming to cultivate adaptable methodologies and pedagogical approaches that mitigate levels of stress^38^. However, the following regression model examined the correlation among all variables while considering the influence of gender and BMI. The present investigation did not exhibit any statistically significant differences between academic stress and diet, contrasting with the findings reported in the previous study^37^.

The analysis revealed a significant finding, demonstrating a statistically significant direct negative relationship between physical activity and academic burnout. This finding is consistent with previous literature that has established an inverse correlation between these two factors. The literature indicates that there is a reduction in levels of anxiety and stress among individuals who engage in regular physical activity^39,40^. The efficacy of physical activity in reducing energy levels, providing a release for frustration, and alleviating muscle tensions is well documented^41^. Furthermore, it has been found to elevate endorphin levels, known as the "happiness hormone", as well as cortisol and norepinephrine levels, both are associated with mental disorders^42^. Furthermore, it has been demonstrated that physical activity facilitates the amelioration of depressive symptoms and the regulation of intricate emotional mechanisms^43^. Balanced eating habits play an important role in well-being and stress management among individuals, especially students. Healthy eating patterns, characterized by healthy eating rich in nutrients, vitamins, and minerals, have been correlated with better mental well-being. Proper nutrition may mitigate the effects of academic burnout, potentially interacting with the influence of physical activity. Research suggests that increased physical activity is potentially associated with a healthier BMI and improved body composition. Maintaining a healthy weight and a healthy diet can positively impact mental well-being and stress management, which may indirectly affect academic stress.

No nation has been able to effectively halt or counteract the increasing rate of weight, because it has been documented to increase consistently over the past 40 years. The notion of thermodynamics is frequently applied to provide a fundamental description of gaining weight, where weight profit or loss is equal to the energy in minus energy out. When interventions halt the weight increase, both of these processes appear to be functioning unfavorably. On the contrary, improving standards of living and well-being are linked to a rise in sedentary lifestyles, a tendency that is also seen in low-income nations and is typified by urbanization, a shift in food culture, and a decrease in quiet time.

### Public health implications

These findings have significant public health ramifications because they draw attention to the growing prevalence of overweight and obesity among college students over the recent decade. Because this group is unable to meet the prescribed amount of weekly exercise frequency, duration, and intensity. Our findings have clear ramifications both from a public health perspective and for student societies and educational settings. Our findings suggest that these organizations and establishments should move swifter in encouraging and facilitating their students' participation in a variety of sports and physical activities. There exists a paucity of evidence regarding the efficacy of extensive awareness campaigns in promoting increased physical activity among the general public. However, research suggests that personalized media messages in various formats have the potential to effectively raise awareness, enhance knowledge, and motivate individuals to engage in higher levels of physical activity.

In 2010, the WHO issued a set of guidelines to inform national policymakers about the necessary levels of physical activity required to prevent noncommunicable diseases, such as overweight and obesity, in light of concerning trends of physical inactivity. The study's results indicate that a significant proportion of young adults continue to not adhere to these guidelines and that the guidelines themselves may not be adequate in addressing the issue of obesity on a population level. The aforementioned predicament emphasizes the necessity for a more comprehensive strategy, and the WHO has recently initiated a worldwide action plan on physical activity spanning from 2018 to 2030 intending to promote increased levels of physical activity among individuals^44^.

### Limitations

There are several limitations to consider when interpreting the results of our investigation. First and foremost, it is important to note that the study's cross-sectional design poses a restriction in that causal interactions were not substantiated. It would be beneficial to draw attention to the adoption of BMI as an overall health determinant, as opposed to body composition metrics like lean and fat mass. However, the main shortcoming of this study is that it did not consider some factors that can influence stress levels, such as family income or health level. Potential next directions for this research include doing a similar analysis with a broader population of university students. To acquire accurate body composition and BMI results, it is also recommended to utilize a bioimpedance instrument. To further enhance data collection, the use of equipment to monitor physical activity would be crucial since it would yield more accurate estimates of the individuals' activity levels.

## Conclusions

The present study demonstrates that a significant ratio of students do not adhere to global exercise guidelines and that the prevalence of overweight individuals is on the rise among both males and females in various age brackets. Hence, interventions focused on reducing academic burnout through physical activity may be enhanced by employing a multifaceted approach that therapeutically addresses the promotion of adherence to the MD and attainment of a healthier BMI. An imperative demand exists for a comprehensive strategy to effect a fundamental change in promoting the increased engagement of college and university students, a responsibility shared among governmental and educational organizations, as well as student welfare associations. Further research is required to investigate the potential mediating impacts of adherence to the MD and BMI in the relationship between physical activity and academic burnout within more diverse populations.

## Data Availability

The raw data supporting the conclusions of this article will be made available by the authors upon reasonable request.
